# Antibody-Mediated Rejection: An Evolving Entity in Heart Transplantation

**DOI:** 10.1155/2012/210210

**Published:** 2012-03-26

**Authors:** Sharon Chih, Andrzej Chruscinski, Heather J. Ross, Kathryn Tinckam, Jagdish Butany, Vivek Rao

**Affiliations:** ^1^Division of Cardiology, University Health Network, Toronto General Hospital, Toronto, ON, Canada M5G 2C4; ^2^Multi-Organ Transplant, University Health Network, Toronto General Hospital, Toronto, ON, Canada M5G 2N2; ^3^Histocompatibility Laboratory, University Health Network, Toronto General Hospital, Toronto, ON, Canada M5G 2M1; ^4^Department of Pathology, University Health Network, Toronto General Hospital, Toronto, ON, Canada M5G 2C4; ^5^Division of Cardiac Surgery, University Health Network, Toronto General Hospital, Toronto, ON, Canada M5G 2C4

## Abstract

Antibody-mediated rejection (AMR) is gaining increasing recognition as a major complication after heart transplantation, posing a significant risk for allograft failure, cardiac allograft vasculopathy, and poor survival. AMR results from activation of the humoral immune arm and the production of donor-specific antibodies (DSA) that bind to the cardiac allograft causing myocardial injury predominantly through complement activation. The diagnosis of AMR has evolved from a clinical diagnosis involving allograft dysfunction and the presence of DSA to a primarily pathologic diagnosis based on histopathology and immunopathology. Treatment for AMR is multifaceted, targeting inhibition of the humoral immune system at different levels with emerging agents including proteasome and complement inhibitors showing particular promise. While there have been significant advances in our current understanding of the pathogenesis, diagnosis, and treatment of AMR, further research is required to determine optimal diagnostic tools, therapeutic agents, and timing of treatment.

## 1. Introduction

Antibody-mediated rejection (AMR) is a diagnostic and therapeutic challenge in human heart transplantation. Although the true incidence of AMR is unknown, it has been reported in 10–20% of patients after heart transplant, typically occurring within a few months after transplant [1, 2]. Late occurrences are, however, not uncommon with one study reporting 25% of AMR cases occurring more than one year after transplantation [1]. A diagnosis of AMR portends a poorer prognosis with an increased incidence of allograft dysfunction, mortality, and cardiac allograft vasculopathy (CAV) [3].

AMR was first described as a clinical entity in 1987 by Herskowitz et al. who identified a subset of heart transplant patients with arteriolar vasculitis and poor outcomes [4]. Hammond et al. subsequently showed that vascular rejection was associated with antibody deposition and complement activation [5]. In 2005, the International Society for Heart and Lung Transplant (ISHLT) published specific guidelines for the diagnosis of AMR [6]. An updated consensus was released in 2011, including a separate companion document detailing the working formulation for the pathologic diagnosis of AMR [7, 8]. This paper will discuss the current understanding of AMR, focussing on pathogenesis, diagnosis, and treatment.

## 2. Pathogenesis

AMR occurs due to a humoral immune response with antibodies binding to endothelium on the transplanted heart [5]. The antibodies are typically directed against human leukocyte antigen (HLA) class I or class II molecules. Antibodies reactive against donor HLA molecules are termed donor-specific antibodies (DSA). These may be preformed and present prior to transplantation or arise de novo after transplantation. The importance of non-donor-specific HLA antibodies arising de novo after transplant is unclear, but may be relevant as they potentially indicate an increased risk for humoral activation. Risk factors for AMR include recipient female sex, multiparity, prior blood transfusions, retransplantation, positive perioperative T-cell flow cytometry crossmatch, elevated panel-reactive antibodies, and prior ventricular assist device [1, 3]. These factors, in common, reflect enhanced humoral responses to antigens and the development of DSA.

DSA binding to the allograft causes myocardial injury and allograft dysfunction predominantly through immune complex activation of the classical pathway of the complement cascade [9]. Antigen-antibody complexes bind to C1q, and in a series of amplified steps, terminal complement components form the membrane attack complex leading to target cell lysis. Complement activation without cell lysis can result in endothelial activation promoting further inflammation [10]. Active complement fragments, C3a and C5a exert direct effects on endothelial cells and are also chemotactic, recruiting neutrophils and macrophages [9, 11]. The split products C4d and C3d are formed during complement activation and covalently bind to protein targets [12]. C4d and C3d have therefore been used as surrogate markers of complement activation.

Anti-HLA antibody binding may also lead to endothelial cell activation by complement independent mechanisms. Direct cross-linking of HLA molecules on the cell surface can activate endothelial cells and lead to the production of growth factors such as fibroblast growth factor, platelet-derived growth factor, monocyte chemotactic protein as well as cytokines and adhesion molecules [13, 14]. Immune effector cells such as natural killer cells, macrophages and neutrophils may also bind to antibody-bound endothelial cells via Fc receptors [12]. These immune effector cells further enhance the inflammatory milieu through cytotoxic actions and via cytokine release. Thus, both complement and noncomplement fixing DSA may activate and injure endothelial cells, thereby predisposing transplant recipients with AMR to the development of CAV [15–17].

The role of non-HLA antibodies in AMR remains an area of contention. Recently, Nath et al. showed that non-HLA antibodies directed against cardiac myosin and vimentin were elevated in heart transplant recipients who subsequently developed AMR and CAV [18]. The appearance of DSA preceded the appearance of non-HLA antibodies. The authors concluded that both allo- and auto-immune mechanisms are likely important in the pathogenesis of AMR and CAV. Non-HLA antibodies to collagen-V and Ka1-tubulin have also been shown to correlate with the development of DSA in heart transplant recipients diagnosed with AMR [19]. Non-HLA antibodies likely damage the allograft through both complement dependent and independent pathways. Antibodies to MICA, however, have not been shown to correlate with rejection episodes, survival, and CAV following heart transplantation [20]. In this study, DSA was confirmed to be an independent risk factor for poor allograft survival, but MICA antibodies did not affect transplant outcomes. The precise role of non-HLA antibodies in AMR remains unclear and more importantly, whether routine detection of these antibodies will impact on the diagnosis of AMR is unknown.

## 3. Diagnosis

The diagnostic criteria for AMR have undergone significant revision. A key difference between the 2005 and 2011 ISHLT guidelines is the proposed shift from a clinical to pathologic diagnosis. The 2005 guidelines recommended diagnosis of AMR based on interpretation of pathologic changes in conjunction with cardiac allograft dysfunction and/or hemodynamic compromise and the presence of DSA [6]. AMR has since been recognized to exist along a spectrum from an asymptomatic phase with occurrence of DSA in isolation to a symptomatic phase with allograft dysfunction and hemodynamic compromise. This is a clinically useful distinction as asymptomatic AMR has been shown to be associated with poorer outcomes [2, 21]. Furthermore, a universally accepted definition for allograft dysfunction does not exist. Allograft dysfunction has been variably described as symptomatic heart failure, left ventricular dysfunction as well as hemodynamic evidence of increased cardiac filling pressures and low cardiac output. Proposed criteria defining allograft dysfunction is shown in Table 1. Importantly, it should be recognized that clinical and hemodynamic parameters, even in the presence of preserved ejection fraction, may also indicate allograft dysfunction.

Screening for DSA is an important component of immune surveillance following heart transplantation. Screening is typically performed with solid phase assays using recombinant HLA-protein-coated beads [22]. Detection of DSA does not, however, confirm the diagnosis of AMR. Circulating DSA may not inflict allograft injury, existing as part of a immunologic process known as accommodation. Accommodation refers to normal allograft function and the absence of antibody-mediated injury despite the presence of circulating DSA [23]. Postulated mechanisms for accommodation include loss of antigens on the allograft, upregulation of cytoprotective molecules, and class switching of antibodies to noncomplement activating subclasses [23]. Another caveat in screening for DSA is that AMR is not excluded by the absence of DSA. In biopsy-proven AMR, DSA may be entirely bound to the allograft and undetectable in the circulation. As an example, Bocrie et al., demonstrated the presence of DSA in eluates from 58.3% of renal allografts with chronic allograft nephropathy but only detectable in the peripheral blood in 16.6% of cases [24]. Thus, current 2011 ISHLT guidelines focus on pathologic features of AMR with demonstration of DSA supportive, but not essential for diagnosis.

### 3.1. Histopathology

Histologic features of AMR as defined by the 2005 ISHLT guidelines include “myocardial injury with endothelial cell swelling and intravascular macrophage accumulation.” Additional features include “interstitial edema and hemorrhage” as well as “intravascular thrombi and myocyte necrosis” [6]. Importantly, many of these features may only be observed in severe cases of AMR with early or less severe cases displaying fewer histologic changes. Several groups have confirmed poor accuracy of isolated histologic features to diagnose AMR. In a study of 3,170 biopsies, Hammond et al. demonstrated a low sensitivity of 63% for endothelial swelling and 30% for vascular adherence of macrophages in the diagnosis of AMR [25]. In a more recent study, morphometric features of early or subclinical AMR lacked sufficient sensitivity when compared to more sensitive detection stains such as C4d [26].

### 3.2. Immunopathology

Detection of AMR may be improved by immunopathologic examination of endomyocardial biopsy specimens. The 2005 ISHLT guidelines recommended immunofluorescence or immunohistochemical staining on frozen and paraffin sections, respectively. Immunofluorescence stains detect immunoglobulin and complement (C3d, C4d, and/or C1q) deposition within capillaries, while immunohistochemistry stains detect capillary CD68 positive macrophages and C4d deposition [6]. The 2011 ISHLT guidelines expanded the recommended immunopathologic panel to include HLA for assessment of capillary integrity, immunoglobulins and fibrin by immunofluorescence and C3d, vascular markers CD34 and CD31, as well as CD3 and CD20 by paraffin immunohistochemistry [7].

C4d deposition has been evaluated in multiple studies as a potential marker for AMR. Gupta et al. evaluated the routine use of C4d immunofluorescence on endomyocardial biopsies after transplant and found that positive C4d staining correlated with a positive retrospective crossmatch and the presence of DSA [27]. Rodriguez et al. demonstrated that C4d coupled with C3d immunofluorescence correlated with a clinical diagnosis of AMR [28]. Interestingly, they found that Immunoglobulin and C1q staining provided no incremental benefit for AMR diagnosis. Immunoglobulins were frequently detected and associated with a staining pattern that did not correlate with the pattern of complement deposition. The use of combined C3d and C4d immunofluorescence staining to detect AMR was also evaluated by Tan et al. who found that this combination could reliably detect AMR defined by the presence of DSA and graft dysfunction [29]. Future studies are needed to confirm a role for C3d staining in addition to C4d in detecting AMR and to determine if immunofluorescence and paraffin immunohistochemistry detection techniques for C3d are equivalent. 

**Figure 1:**
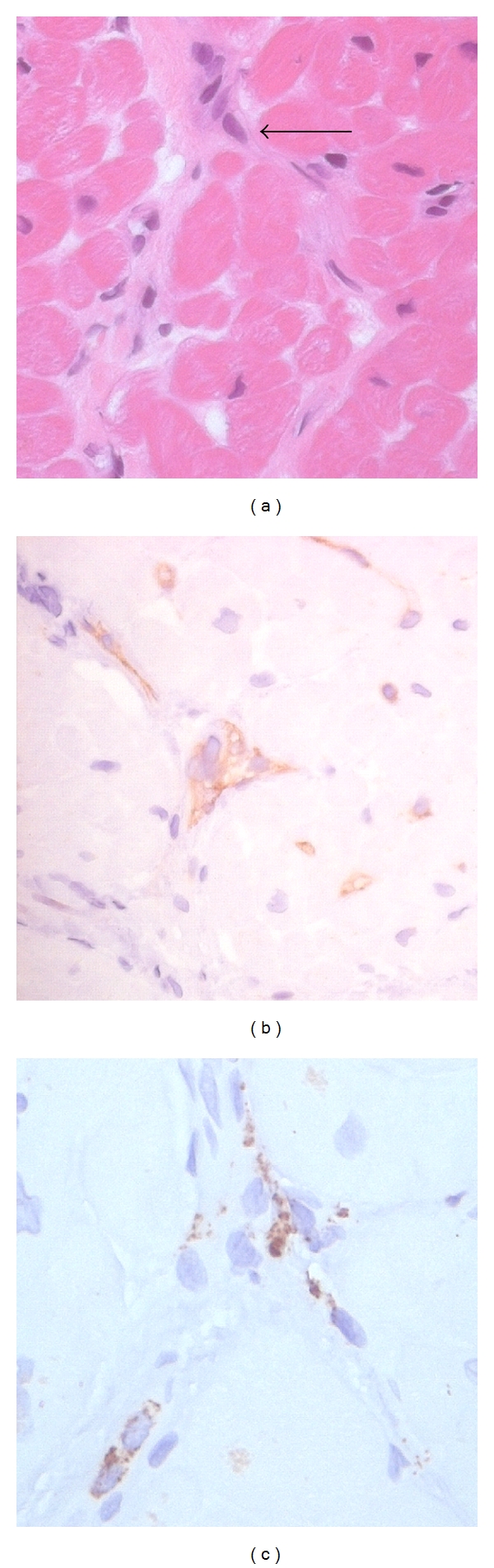
Histopathologic and immunopathologic biopsy features of AMR. (a) A biopsy sample stained with hematoxylin and eosin shows evidence of endothelial cell swelling (arrow). (b) An immunoperoxidase stain shows diffuse C4d deposition in capillaries. (c) An immunoperoxidase stain confirms the presence of intravascular macrophages with positive staining for CD68.

A potentially useful marker of AMR is CD68, a cell surface antigen present on macrophages. Rodriguez et al. showed that patients with graft dysfunction likely related to AMR had positive staining for intravascular macrophages while patients with preserved graft function did not display CD68 staining [28]. Several studies have demonstrated a correlation between the presence of intravascular macrophages and AMR [30, 31]. CD68 positivity may identify a more severe form of AMR associated with allograft dysfunction, and therefore early AMR may not be detected with this stain. Further work is necessary to determine antigen staining combinations sensitive to and specific for AMR that can also detect early damage due to antibody deposition.  Figure 1 shows examples of histopathologic and immunopathologic biopsy findings of AMR in heart transplantation.

### 3.3. ISHLT Guidelines

The ISHLT recently proposed a new AMR grading scheme that is focussed on a shift towards a pathologic diagnosis [8]. The proposed AMR grading scheme incorporates an AMR severity scale from pAMR 0 to pAMR 3 (“p” reflecting a pathologic-based diagnosis). A biopsy with pAMR 0 has neither histopathologic nor immunopathologic features of AMR. A biopsy with pAMR 1 has either histopathologic or immunopathologic features. A biopsy with pAMR 2 has both histopathologic and immunopathologic features. Finally, a biopsy with pAMR 3 has severe pathologic features of AMR such as interstitial hemorrhage and significant edema. Similar to the current grading scheme for cell-mediated rejection, associated clinical findings such as “hemodynamic compromise” may assist but are not required for diagnosis.

A proposed timeline for AMR screening is also proposed in the new ISHLT guidelines [7]. The guidelines recommend that all biopsies be routinely evaluated for histopathologic evidence of AMR. Immunopathologic testing on biopsies in the first year is recommended at 2 weeks and at 1, 3, 6, and 12 months after transplant. Interval immunopathologic testing is recommended if there is occurrence of histologic, serologic, or clinical suspicion of AMR. The guidelines also recommend that following a positive biopsy result, repeat testing should be performed on subsequent biopsies until a negative result is obtained. DSA is an important monitoring tool for AMR, and the ISHLT recommends testing with either solid phase- or cell-based assays at 2 weeks and at 1, 3, 6, and 12 months after heart transplant and annually thereafter, in the absence of clinical suspicion.

### 3.4. Future Directions for Diagnosis

There has been growing interest in the use of microarray technology to monitor gene expression and diagnose AMR. Microarrays were used to study endothelial-associated gene transcripts (ENDATs) in kidney allograft biopsies [32, 33]. In comparison to C4d, ENDAT expression showed improved accuracy for predicting graft loss in DSA positive patients: 77% sensitivity for ENDAT versus 31% for C4d. The authors suggest that non-complement fixing DSA may potentially be as damaging to endothelial and graft function as complement fixing DSA. The combination of DSA and ENDAT expression thus identifies a population of patients with active antibody mediated damage to endothelial cells and a poor prognosis. This type of molecular screening tool may prove useful in detecting both early stages of AMR and also AMR due to non-complement fixing DSA. At present, it is unclear if similar measurements can be made in cardiac biopsies and if they will provide additional predictive value in diagnosing AMR compared to stains such as C4d.

## 4. Management

The development of evidence-based guidelines for the management of AMR in heart transplantation is greatly needed. Persisting controversy over the diagnostic criteria for AMR, continued development of diagnostic techniques and a paucity of robust data to substantiate traditional therapies represent important challenges. At present, the management of AMR is nonstandardized, and optimal surveillance, treatment, and timing of intervention are undefined. AMR is difficult to treat, and therapy remains empiric with supporting evidence limited to small, nonrandomized studies with short followup. Furthermore, although it is widely accepted that AMR associated with allograft dysfunction should be aggressively treated, the management of asymptomatic AMR is less clear. While close surveillance is advocated for this group of patients, the ideal frequency and mode of assessment including evaluation of allograft function, performance of endomyocardial biopsies, and HLA antibody screening are unknown. Additionally, although studies suggest that asymptomatic AMR is associated with reduced patient survival, and an increased risk for development of CAV, implementation of surveillance and treatment has not been proven to improve outcomes [2, 21].

**Figure 2:**
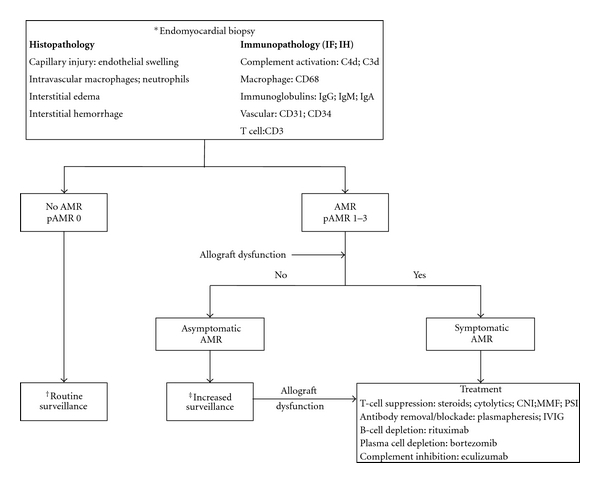
Proposed management algorithm for AMR in heart transplantation. AMR: antibody-mediated rejection, CNI: calcineurin inhibitors, DSA: donor specific antibodies, IF: immunofluorescence, IH: immunohistochemistry, IVIG: intravenous immunoglobulin, MMF: mycophenolate mofetil, pAMR: ISHLT pathologic AMR grade 0–3, and PSI: proliferation signal inhibitor. *Endomyocardial biopsy frequency as per institution protocol. ^†^DSA monitoring at week 2, months 1, 3, 6, and 12 in first year after transplant and annually thereafter. ^‡^Assessment of allograft function every month and DSA every 3 months until two negative or unchanged results.

**Figure 3:**
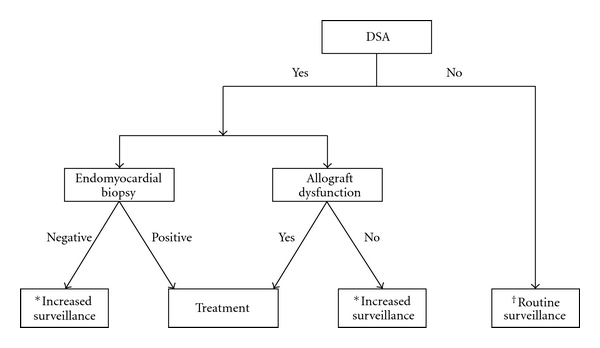
Proposed DSA treatment algorithm. AMR: antibody-mediated rejection, DSA: donor-specific antibodies. *Assessment of allograft function every month and DSA every 3 months until two negative or unchanged results. ^†^DSA monitoring at week 2, months 1, 3, 6, and 12 in first year after transplant and annually thereafter.

A proposed management algorithm for AMR is summarized in Figure 2. Incorporating the recent 2011 ISHLT guidelines, AMR is diagnosed by identifying specific histopathologic and/or immunopathologic features on endomyocardial biopsy. Treatment is recommended for symptomatic AMR, although less certain for asymptomatic AMR. Allograft dysfunction, based on clinical, hemodynamic and imaging parameters differentiates symptomatic from asymptomatic AMR (Table 1). Routine posttransplant surveillance for AMR should include pathologic evaluation on endomyocardial biopsy, assessment of allograft function, and monitoring for DSA. In individuals with pathologic evidence of AMR, increased frequency of surveillance is recommended with assessment of allograft function every month and DSA monitoring every three months until two negative or unchanged results (Figures 2 and 3).

A potential DSA monitoring algorithm is shown in Figure 3. Individuals with DSA should be evaluated for AMR with endomyocardial biopsy and allograft function assessment. De novo DSA, especially those that persist, is an independent predictor of poor survival after transplant [34]. Thus, although not proven to improve outcomes, treatment may be considered in patients with asymptomatic AMR who develop DSA. The presence of DSA associated with new and otherwise unexplained allograft dysfunction raises the suspicion of AMR and consideration for treatment. As DSA may exist without causing allograft injury (accommodation), their detection in the circulation in isolation without allograft dysfunction or biopsy features of AMR may not require treatment. The presence of DSA should prompt increased AMR surveillance. Additionally, increased AMR surveillance is also recommended in individuals who develop de novo non-HLA antibodies due to the potential risk of heightened humoral activation.

## 5. Treatment

Altered humoral immunity with plasma cell production of antibodies directed against donor antigens on the allograft is primarily responsible for myocardial injury in AMR. Plasma cells are generated by T-lymphocyte-dependent activation and generation of memory B-lymphocytes. Treatment strategies for AMR are directed at inhibiting the humoral response at various levels by targeting (1) removal and blockade of circulating antibodies, (2) depletion of B-lymphocytes, (3) depletion of plasma cells, (4) suppression of T-lymphocyte dependent antibody responses, and (5) inhibition of the complement cascade. While several agents have been used successfully and others show promise, AMR remains difficult to treat and is often refractory to therapy.

### 5.1. T-Lymphocyte Suppression

B-lymphocyte responses, including B-lymphocyte activation, the generation of memory B-lymphocytes, and high-affinity antibodies are dependent on T-lymphocytes. Suppression of T-lymphocytes, therefore, indirectly inhibits B-lymphocytes. Cytolytic antibodies (thymoglobulin; OKT3), corticosteroids, calcineurin inhibitors (cyclosporine; tacrolimus), mycophenolate mofetil, and proliferation signal inhibitors (sirolimus; everolimus) inhibit T-cell proliferation and/or activation through a variety of mechanisms and may be used to treat AMR. Many of these drugs also exhibit direct effects on B-lymphocytes. For example, thymoglobulin is a polyclonal antibody containing multiple anti-T-lymphocyte antibodies that target classes I and II, HLA molecules, associated ligands (CD3, CD4, and CD8), costimulatory molecules, cell adhesion molecules as well as surface molecules expressed on B-lymphocytes (CD20) and plasma cells (CD38 and CD138) [35]. Calcineurin inhibitors have antiproliferative and proapoptotic effects on activated B-lymphocytes. Mycophenolate mofetil may lead to hypogammaglobulinemia, and there is in vitro evidence that it plays a direct role in inhibiting B-lymphocytes [36, 37]. The efficacy of single or combination immunosuppression therapy for the prevention and/or treatment of AMR has not been evaluated. Baseline immunosuppression is usually augmented after AMR.

### 5.2. Plasmapheresis

Plasmapheresis removes circulating plasma antibodies by extracorporeal separation of plasma from cellular blood components using either centrifugation or membrane filtration. As antibody production is unaffected, antibody levels may increase after discontinuation of plasmapheresis. Potential adverse effects include complications related to vascular access, hypotension due intravascular volume contraction, bleeding from depletion of clotting factors as well as allergic reactions and blood-borne infection from plasma repletion with replacement fluids such as albumin and fresh frozen plasma.

In 2001, Grauhan et al. published the first clinical case series of plasmapheresis for the treatment of AMR after heart transplantation [38]. Between 1986 and 1999, 18 episodes of AMR with associated hemodynamic compromise involving 13 patients were identified. All patients received treatment with corticosteroids and cytolytic antibodies with the addition of plasmapheresis in six patients. All six patients treated with plasmapheresis survived, while only two of seven patients in the nonplasmapheresis group survived. The authors concluded that a randomized controlled study was justified; however, only small case series (*n* < 15) followed. Although these small studies have consistently demonstrated that plasmapheresis is an effective treatment for AMR, variation in protocols with differing duration and frequency of treatment and adjunctive treatment confounds interpretation. Similarly, an ideal plasmapheresis treatment regimen has not been established with the number of treatments in studies varying from days to weeks.

### 5.3. Intravenous Immunoglobulin

Intravenous immunoglobulin (IVIG) is comprised of 90% IgG, extracted from pooled plasma from between 50,000 and 100,000 blood donors [39]. A number of immunomodulatory effects have been described for IVIG including blockade of Fc receptors, complement inhibition, and downregulation of B-lymphocyte receptors [39]. IVIG is used as standard therapy for primary immune deficiencies, autoimmune and inflammatory disorders. Various IVIG preparations are available, differing in IgG concentration, osmolarity, and sodium and sugar content. IVIG is generally well tolerated with a <5% incidence of adverse reactions including mild infusion-related effects (usually first dose) that generally improve with temporary cessation or reduction in infusion rate, anaphylactic reactions associated with IgA sensitization in patients with IgA deficiency, thrombosis, and volume overload. The latter is due to an average volume requirement of 1 to 1.5 L (treatment dose 1-2 g/kg) and sodium load for drug administration, and poses difficulties for its application in patients with cardiac AMR with concurrent allograft dysfunction and heart failure.

Experience with IVIG in transplantation is largely derived from renal transplantation. In this patient population, IVIG has mostly been evaluated for desensitization and shown to reduce PRA and increase conversion to a negative complement dependent cytotoxic crossmatch [40]. Recently, Montgomery et al. reported superior patient survival for sensitized individuals undergoing desensitization with IVIG and plasmapheresis prior to transplant in comparison to patients undergoing an HLA-matched transplant [41]. IVIG has also been used successfully to treat AMR after renal transplant, usually in combination with plasmapheresis and rituximab [42–44]. Data from large case series are, however, not available, and IVIG has not been systematically evaluated for the treatment of AMR after heart transplant. Jordan et al. were the first to describe the successful treatment of AMR after heart transplant with IVIG. Three cardiac and seven renal transplant patients with severe AMR were treated with IVIG [45]. All cardiac patients had reversal of AMR and survived to six months. All seven renal patients recovered with no recurrent events. All ten patients demonstrated a rapid reduction in DSA.

### 5.4. Rituximab

Rituximab is a chimeric monoclonal IgG antibody, comprised of human constant regions and mouse variable regions directed against the CD20 pan-B lymphocyte surface molecule. Rituximab depletes B-lymphocytes by antibody-dependent cell-mediated cytotoxicity, complement-dependent cytotoxicity, and induction of apoptosis through CD20 cellular crosslinking and caspase activation [46]. Rituximab was originally approved for the treatment of B-cell lymphoma and is now also used for a number of autoimmune diseases including immune thrombocytopenic purpura and myasthenia gravis.

The efficacy of Rituximab for AMR treatment after heart transplant has only been examined in case reports and one small case series (*n* = 8) [47]. The majority found a benefit by demonstrating improved allograft function. Most centres, however, administered rituximab in combination with other treatments, preventing sole evaluation of rituximab. Although the ideal dosing for rituximab is unknown, a commonly used regimen in these and hematologic studies is 375 mg/m^2^/week for up to 4 weeks. Pharmacodynamic studies in patients with lymphoma have shown maximal B-lymphocyte depleting effects at three to four months with low or undetectable numbers in the peripheral blood for up to six months and full recovery within 12 months [48]. Interestingly, it has been demonstrated in patients with end-stage renal disease that a similar reduction in PRA, and B-lymphocyte depletion may be achieved using low (50 mg/m2) or high doses (150 and 375 mg/m2) of rituximab [49]. Furthermore, Fc gamma receptor polymorphisms affecting antibody-dependent cell-mediated cytotoxicity results in variation in rituximab induced B-lymphocyte depletion among individuals, producing further complexity in determining an ideal treatment dosing regimen [50, 51].

The safety and tolerability of rituximab is well established in the context of hematologic malignancies. Mild-to-moderate first dose infusion-related reaction with fever and hives account for the majority of adverse effects. Concerns have, however, been raised over an increased risk of serious infections with rituximab therapy after transplantation. In a study of 22 renal transplant recipients, rituximab was shown to be effective in treating AMR but associated with a high incidence of infection [52]. Eighty-six percent of patients experienced serious infection including three cases of septic shock. It is probable that cumulative immunosuppression was intense and contributed to the development of infection as rituximab was administered in combination with six sessions of plasmapheresis, pulse steroids, tacrolimus, mycophenolate mofetil as well as cytolytic antibodies in a proportion of patients.

### 5.5. Bortezomib

Proteasomes are responsible for the processing and degradation of ubiquitin-labeled proteins. Bortezomib is a selective 26S proteasome inhibitor depleting plasma cells and antibody production by inducing apoptosis via cell cycle arrest as well as inducing the unfolded protein response due to increased endoplasmic reticulum stress from accumulation of misfolded proteins [53, 54]. Additional immunomodulatory effects of bortezomib include gene transcriptional activator nuclear factor kappa B inhibition and inhibition of antigen processing by preventing peptide generation and MHC class I expression [53, 54].

Bortezomib is currently approved for the treatment of multiple myeloma. Small studies in renal transplant patients have shown that bortezomib effectively decreases HLA antibodies in patients with stable renal function and is effective in treating refractory AMR [55–59]. Woodle et al. recently presented results from the largest multicentre study of 107 cases of AMR in 91 solid organ transplant recipients [60]. The patient population comprised predominantly of kidney transplant recipients (*n* = 81), but also included five heart transplant recipients. Bortezomib was administered in four doses, each preceded by plasmapheresis, and all patients also received a single dose of rituximab. Over an average 9 month followup, 58% of patients showed histologic improvement, with 81% and 96% graft and patient survival, respectively. Additionally, there was a >50% reduction in DSA in 40% of patients at 14 days after treatment. An 11.2% incidence of opportunistic infection was reported. Studies in transplantation and multiple myeloma series have reported high tolerability of Bortezomib with common adverse effects including fatigue, gastrointestinal toxicity, thrombocytopenia, neutropenia, and peripheral neuropathy.

### 5.6. Eculizumab

Eculizumab is a humanized monoclonal antibody directed against C5, thereby preventing complement activation by inhibiting cleavage of C5 to C5a and C5b, and formation of the membrane attack complex. Eculizumab is currently approved in the United States for the treatment of paroxysmal nocturnal hemoglobinuria. Experimental studies in sensitized mice have shown that eculizumab as part of a multi-immunosuppressive regimen prevents allograft rejection and improves allograft survival [61]. At present, clinical studies of eculizumab in AMR are observational and preliminary. Locke et al. reported the successful treatment of a renal transplant recipient with refractory AMR using eculizumab [62]. In another study of highly sensitized renal transplant patients with DSA, a significant reduction in incidence of early AMR at <3 months was demonstrated for eculizumab (6.25% incidence of AMR for eculizumab treated group compared to 40% for the historical control group) [63].

## 6. Conclusion

The diagnosis and management of AMR in heart transplantation has evolved significantly over the last decade. Current international multidisciplinary consensus recommend a pathologic-based diagnosis, combining specific histologic and immunopathologic features on endomyocardial biopsy and utilizing accompanying allograft dysfunction and/or circulating DSA as additional supporting evidence. AMR as a disease entity will continue to evolve and become better defined with advances in diagnostic techniques and improved understanding of key pathogenic molecular and immunologic mechanisms. Current available therapeutic options modify the humoral immune response by targeting the removal and blockade of antibodies, and the depletion of T- and B-lymphocytes. Emerging therapies showing promise include agents that directly deplete plasma cells and antibody production, such as bortezomib, as well as complement inhibiting agents such as eculizumab. The scarcity of heart transplantation as a clinical resource and current poor patient and allograft outcomes associated with AMR provide significant impetus for the development of effective treatment strategies. It is anticipated that the 2011 ISHLT consensus guidelines on AMR diagnosis and management will further facilitate the goal of developing a standardized evidence based management approach to AMR in heart transplantation.

## Figures and Tables

**Table 1:** Definition of allograft dysfunction (≥1 criteria required).

Clinical heart failure	Symptoms and signs of low cardiac output and/or pulmonary or systemic congestion
Hemodynamics	PCWP > 20 mm Hg, and CI < 2.0 L/min/m^2^

Inotropes	Requirement for inotropic drugs

Restrictive physiology	Echocardiogram: LVEF >50%, E to A ratio >2, IVRT <60 ms and DT <150 ms
	*Or *
	Right heart catheterization: RAP >12 mm Hg, PCWP >25 mm Hg and CI < 2.0 L/min/m^2^

Systolic dysfunction	LVEF ≤45% or FS ≤20% or ≥25% decrease of LVEF or FS from baseline

A: A-wave, CI: cardiac index, DT: deceleration time, E: E-wave, FS: fractional shortening, IVRT: isovolumic relaxation time, LVEF: left ventricular ejection fraction, PCWP: pulmonary capillary wedge pressure, and RAP: right atrial pressure.
